# The effectiveness of a digital game to improve public perception of dementia: A pretest-posttest evaluation

**DOI:** 10.1371/journal.pone.0257337

**Published:** 2021-10-08

**Authors:** Gillian Carter, Christine Brown Wilson, Gary Mitchell

**Affiliations:** School of Nursing & Midwifery, Queen’s University Belfast, Belfast, United Kingdom; Texas State University, UNITED STATES

## Abstract

The global impact of dementia is a key healthcare priority, and although it is possible to live well with dementia, public perception is often negative. Serious digital games are becoming a credible delivery method to educate/train individuals in the business and health sectors and to challenge perceptions. The main objective of the study was to evaluate the effectiveness of a digital game prototype on individual attitudes towards dementia. A digital game to improve public knowledge and understanding about dementia (www.dementiagame.com) was co-designed with people living with dementia and student nurses. The Game was evaluated using a pretest-posttest design. Participants for the evaluation were recruited via social media in one UK university and completed the Approaches to Dementia Questionnaire (ADQ) before and after playing the game. Overall, 457 individuals completed both pre and post test questionnaires. The total ADQ score demonstrated a significant improvement in positive attitudes (p < 0.001), and both subscales of Hope and Recognition of Personhood also saw significant improvements (p < 0.001). The use of a serious digital game has demonstrated a significant effect on the respondents’ perceptions of dementia. Overall, there was a more positive view of the abilities of people with dementia and what their capabilities were. They were also more likely to be recognised as unique individuals with the same values as any other person. The benefit of using digital gaming to improve perceptions of dementia has been demonstrated, nonetheless further research is required to reach a more diverse population and test as a Randomised Control Trial to provide definitive evidence for use in policy and practice.

## Introduction

It is well documented that the impact of dementia is a global healthcare priority [1, 2]. In 2015 it was reported that 46.8million people were living with dementia [3], a figure that is expected to double every 20 years [1]. This highlights that not only is the number of people living with dementia increasing but that this will be accompanied by a rise of those affected by dementia such as a family member, friend, healthcare provider or support professional [1, 4].

Dementia is one of the major causes of disability and dependency among older adults worldwide [1, 4], however attitudes and public perceptions of dementia still remain below par. In 2019 Alzheimer’s Disease International (ADI) published results of the attitudes to dementia survey, completed by 70,000 respondents representing 155 countries [5]. Although awareness of dementia is increasing, understanding of the development and progression of the disease remains low. As such, rather than recognising dementia as a neurodegenerative disease, two thirds of all respondents and 62% of healthcare practitioners thought dementia to be caused by normal ageing. In the UK specifically, only 51% of the public recognise the terminal nature of dementia despite it being a leading cause of death [6].

Initiatives to engage the public with lived experiences of dementia, and Dementia Friendly Communities [6, 7] are helping to address negative attitudes, but stigma and discrimination are still experienced by some people living with dementia. Public stigma is generated by stereotypes, and prejudice resulting in discriminatory behaviour towards those with dementia [8]. For example, in South East Asia and Africa, 63% and 67% respectively, of respondents of the ADI survey living with dementia said their symptoms had been ridiculed [5]. Stigma has the potential to negatively impact on the lives of people living with dementia and their carers as they may absorb the public conception of dementia leading to a belief in their own loss of competence or that they should no longer engage in public activities [8]. In order to eradicate such attitudes, the dispelling of myths and the continual improvement of public awareness of dementia is essential.

Serious digital games are a form of a computer-delivered intervention, designed for a purpose other than purely entertainment with the aim of educating or promoting behaviour change [9]. Serious gaming/gamification for health professionals has been demonstrated to be an effective delivery mode of education and on occasion more effective for improving knowledge and skills than traditional methods [10]. A recent meta-analysis of research in which this method was used for healthy lifestyle promotion concluded digital gaming is effective as stand-alone or a multi-component program and appeals to diverse population regardless of age or gender [9].

To tailor a serious digital game to our specific needs of improving public perception of dementia, a prototype of the game (www.dementiagame.com) was developed through co-design with people living with dementia. The aim of this study was to evaluate the effectiveness of the digital game prototype to determine if it improved individual attitudes towards dementia through ‘players’ completing the Approaches to Dementia Questionnaire (ADQ) [11] questionnaire pre and post access to the digital game.

## Methods

### About the game

The ‘Dementia Game’ (www.dementiagame.com) is digital game that challenges stereotypes and stigma around dementia. People living with dementia shared their experiences to identify themes the game would cover [12], then along with nursing students and the research team we co-designed questions and the format of the game in coproduction workshops supported by Focus Games™ (https://focusgames.com/).

**Fig 1:**
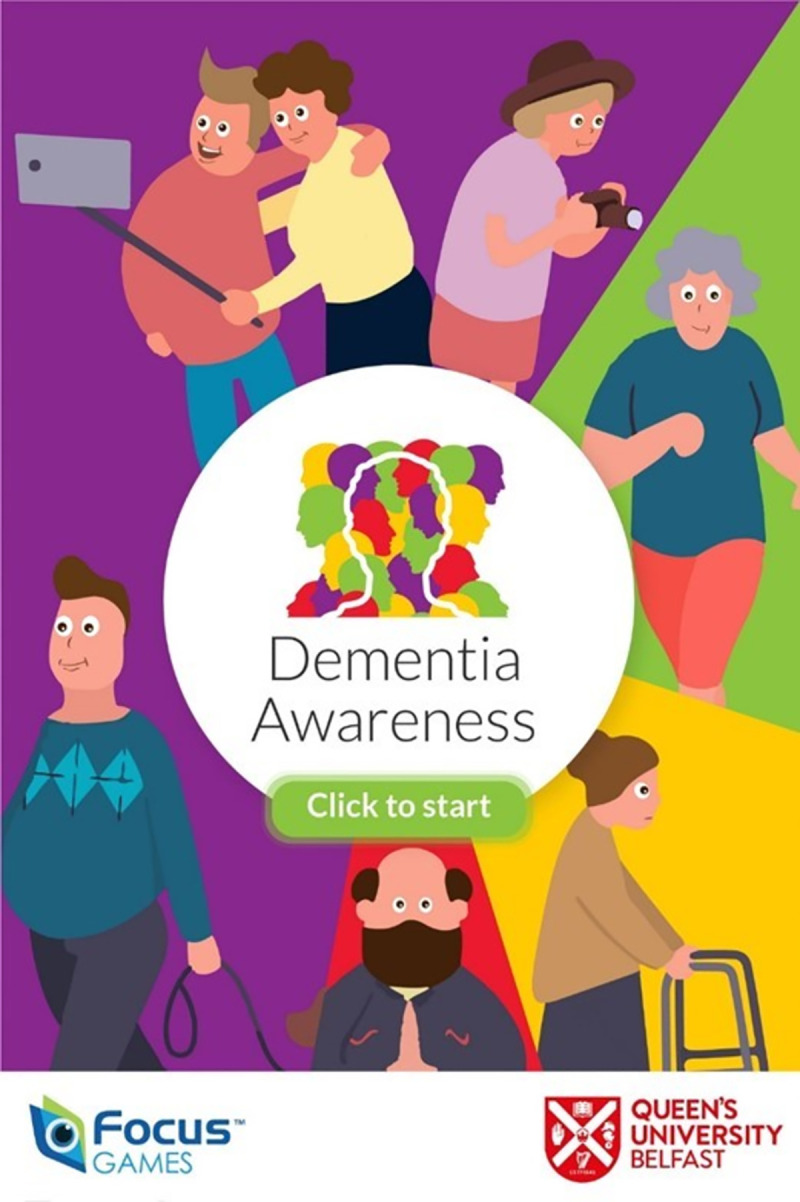
Start screen of dementia awareness game (with kind permission from Focus Games™) accessible from www.dementiagame.com.

**Fig 2:**
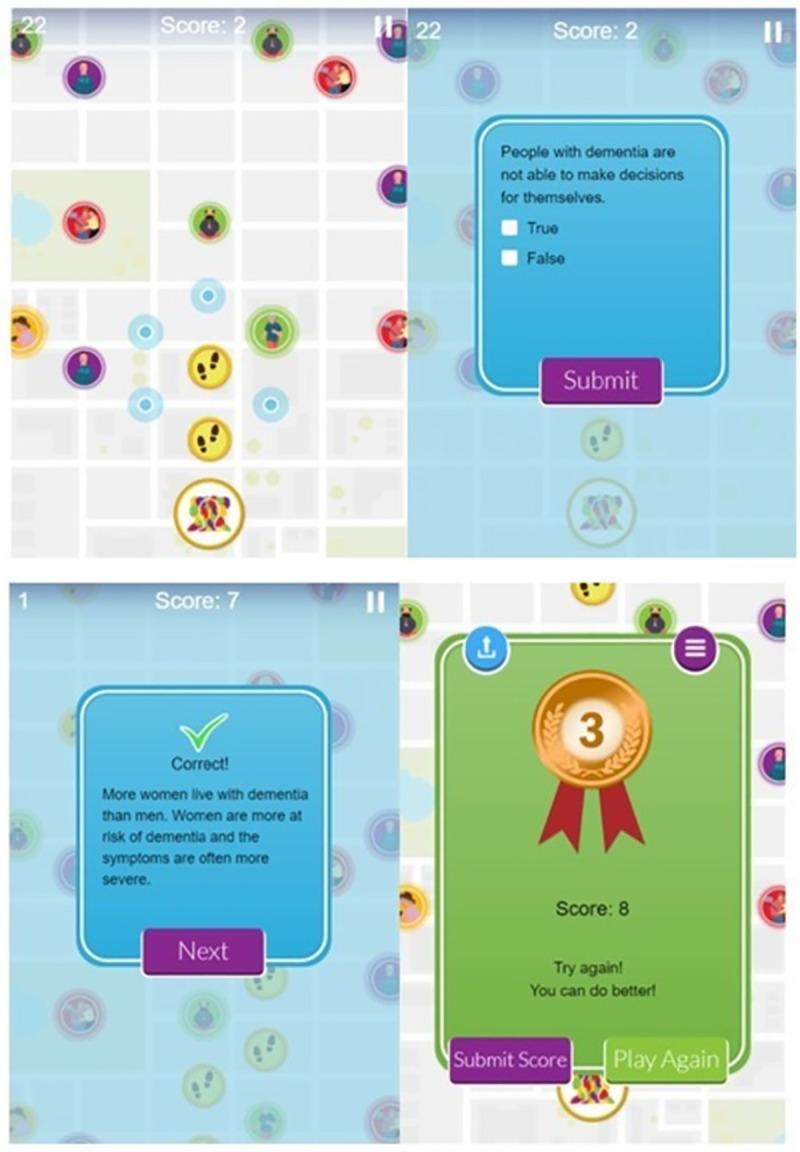
Example screens of dementia awareness game during play (with kind permission from Focus Games™) accessible from www.dementiagame.com.

The ‘Dementia Game’ is a web application (HTML5) which can be played on any device with an internet connection. It is designed to be simple to use, where players must navigate a path to reach the finish line. Players answer a series of questions related to dementia that are provided in a random order from an existing question bank. These questions were co-designed by people living with dementia and were about challenging misconceptions e.g., that dementia is *not* a normal part of ageing, and they also addressed different questions than those within the ADQ. These challenge players’ knowledge about dementia, including their attitude and behaviours. Answering questions correctly wins points with bonus points earned for reaching the finish line. Players can post their scores and challenge friends and colleagues to play. The ‘Dementia Game’ takes approximately 90 seconds to play and players can have multiple attempts (please see Figs 1 and 2).

### Design

A pretest-posttest design was used to determine the effectiveness of the serious digital game. After participants had completed the ADQ [11] provided online at baseline, a link was made available to commence the dementia game. At the end of the game the ADQ was completed again. For this study individuals were requested to play the game and answer the ADQ once, then their IP address was logged, and they would not receive the questionnaire again. After this they were welcome to continue accessing the game.

### Participants

The sample size was calculated using the software G*Power 3.1.9.4 [13, 14]. Assuming p < 0.05 and a power of 0.9, the required number of participants to observe an effect size of 0.5 in the attitudes towards dementia was calculated to be 44 matched pairs. Potential participants were recruited via social media using central university Facebook, Twitter, LinkedIn, and Instagram pages. Information about this study and links to participate were shared weekly on these channels from 01.09.2019 to 30.09.2019. Access to the questionnaires and game closed at the end of this period. Participants did not have to sign written consent forms and consent was implied through their active selection of the game weblink. Participants were required to use their own laptop, computer tablet or mobile phone to access the game and complete the questionnaires. Due to the nature of this study, we did not apply active inclusion or exclusion criteria. Given the recruitment strategy, it is likely that most participants are prospective, current, or past university students.

### Evaluation

The ADQ [11] is a 19-item questionnaire, that has been demonstrated to be valid and reliable [11, 15, 16]. Each item is comprised of a five-point Likert scale measuring the level of agreement or disagreement with a statement, with total scores ranging 19–95. Higher scores indicate more positive attitudes towards people with dementia. It is also comprised of two subscales ‘*Recognition of Personhood’* and *‘Hope’*. The subscale on person centeredness focuses on the way a person with dementia is seen as an individual person with the capabilities whilst the subscale for Hope demonstrates either an optimistic or pessimistic approach to a person with dementia. For the purpose of this study these helped to understand someone’s attitude towards dementia by determining the extent to which those playing the game recognised people affected by dementia as unique individuals with the same value as any other person, and it also highlighted any sense of optimism or pessimism the person had about the abilities and the future of a person affected by dementia.

General demographic questions were asked at baseline along with three questions related to experiences of knowing someone with dementia, working with those living with dementia, and training/education about dementia [11, 15]. This was to enable us to identify if prior experience or training in dementia care influenced the change (if any) in attitudes towards dementia.

### Analysis

All data were transferred from Survey Monkey to an excel spreadsheet, where they were cleaned, coded and scored according to the ADQ guidelines. Data were quality checked by two members of the research team (GC and GM). Demographic data were reviewed using descriptive analysis. Matched pairs for the ADQ scores at baseline and follow-up were analysed using dependent t-tests, with a two-tailed significance level set at α = .05. The effect size was calculated with Pearson’s correlation *r*, a versatile measure of the strength of an experimental effect [17], constrained to lie between 0 (no effect) and 1 (perfect effect). The effect size is considered small if the value of *r* is around .10, medium if *r* varies around .30 and large if around or greater than .50 [18]. Analysis was conducted using IBM SPSS statistics 21 with advice provided by a statistician independent to the study.

### Ethics

This study received ethical approval by Queen’s University Belfast, School of Nursing and Midwifery Research Ethics Committee in April 2019 (Reference: CBrownWilson 03.19 M2.V1). Participants did not provide verbal or written consent but were informed that they were under no obligation to complete any of the questionnaires. Participants gave their consent to complete the questionnaire when they actively accessed the survey web links.

## Results

In total 997 participants completed the baseline questionnaire and subsequently played the ‘Dementia Game’. Of these 457 people also completed the post test questionnaire. Only the 457 matched pairs were used for analysis. Table 1 provides the demographics of these respondents and their answers to the three dementia related questions posed. Of the 457 respondents, the vast majority were white and female, with 78.1% between the age 18–34. Nearly two-thirds did not have a family member or close friend living with dementia, and just over half had not worked with people living with dementia, and finally 55% had completed some form of training or education about dementia.

**Table 1:** Demographics of respondents who completed both questionnaires (n = 457).

Question	Frequency (%)
What is your age?	
18–24	233 (51.0)
25–34	124 (27.1)
35–44	57 (12.5)
45–54	36 (7.9)
55–64	7 (1.5)
What is your gender?	
Male	29 (6.3)
Female	427 (93.4)
Prefer not to say	1 (0.2)
Which race/ethnicity best describes you?	
White	434 (95.0)
Mixed	5 (1.1)
Asian	15 (3.3)
Black	2 (0.4)
Other	1 (0.2)
I have a family member or close friend living with dementia?	
No	284 (62.1)
Yes	171(37.4)
Did not respond	2 (0.4)
I work with people living with dementia	
No	249 (54.5)
Yes	208 (45.5)
I have completed training/education about dementia	
No	204 (44.6)
Yes	253 (55.4)

Overall, the ADQ total score demonstrated a significant increase from 79.60 (±6.663) pretest to 82.24 (±6.580) posttest (p < .001). Similarly, significant increase in scores were found for the *Hope* and *Recognition of Personhood* subscales (both p < .001) (Table 2). The ADQ total score and subscale scores all yielded significantly large effect sizes (between .592 and.725).

**Table 2:** Paired t-test (n = 457) of pre-game (T0) and post-game (T1) ADQ scores.

	T0 Mean (SD)	T1 Mean (SD)	t	Sig. (2-tailed)^a^	Correlation *r*	Sig. (2-tailed)^a^
ADQ Total score	79.60 (±6.663)	82.24 (±6.580)	-10.372	p < .001	.661	p < .001
ADQ Hope subscale	33.65 (±4.074)	35.34 (±4.325)	-9.522	p < .001	.592	p < .001
ADQ Recognition of Personhood subscale	45.95 (±3.609)	46.90 (±3.341)	-7.859	p < .001	.725	p < .001

ADQ, Approaches to Dementia Questionnaire; SD, Standard deviation

^a^For all analysis, a two-tailed p value of < 0.05 indicated statistical significance

Subgroup analysis of the dichotomous responses to the three personal dementia questions at baseline showed that they all had significant improvement in perceptions whether or not they had personal experience of dementia, worked with people living with dementia or received training in dementia (Table 3).

**Table 3:** Subgroup analysis scores with t-test value and significance level.

	Total ADQ score (SD)	t	Hope Subscale Score (SD)	t	Recognition of Personhood Subscale Score (SD)	t
I have a family member or close friend living with dementia^a^						
No (n = 284)	T_0_ 79.49 (±7.016)	-7.735^b^	T_0_ 33.67 (±4.298)	-7.195^b^	T_0_ 45.82 (±3.750)	-6.270^b^
	T_1_ 82.27 (±6.672)		T_1_ 35.44 (±4.378)		T_1_ 46.82 (±3.425)	
Yes (n = 171)	T_0_ 79.75 (±6.086)	-7.282^b^	T_0_ 33.63 (±3.708)	-6.411^b^	T_0_ 46.12 (±3.375)	-4.647^b^
	T_1_ 82.13 (±6.443)		T_1_ 35.15 (±4.246)		T_1_ 46.98 (±3.207)	
I work with people living with dementia						
No (n = 249)	T_0_ 78.72 (±6.985)	-8.043^b^	T_0_ 33.28 (±4.139)	-7.147^b^	T_0_ 45.44 (±3.774)	-6.520^b^
	T_1_ 81.57 (±6.993)		T_1_ 35.06 (±4.383)		T_1_ 46.51 (±3.627)	
Yes (n = 208)	T_0_ 80.64 (±6.109)	-6.552^b^	T_0_ 34.10 (±3.959)	-6.284^b^	T_0_ 46.55 (±3.311)	-4.519^b^
	T_1_ 83.04 (±5.967)		T_1_ 35.68 (±4.241)		T_1_ 47.36 (±2.904)	
I have completed training/ education about dementia						
No (n = 204)	T_0_ 77.85 (±6.969)	-6.274^b^	T_0_ 32.76 (±4.074)	-5.725^b^	T_0_ 45.09 (±3.780)	-4.926^b^
	T_1_ 80.53 (±7.205)		T_1_ 34.48 (±4.439)		T_1_ 46.05 (±3.749)	
Yes (n = 253)	T_0_ 81.00 (±6.062)	-8.535^b^	T_0_ 34.37 (±3.937)	-7.905^b^	T_0_ 46.64 (±3.315)	-6.198^b^
	T_1_ 83.62 (±5.679)		T_1_ 36.04 (±4.109)		T_1_ 47.58 (±2.797)	

ADQ, Approaches to Dementia Questionnaire; SD, Standard deviation; T_0,_ baseline; T_1,_ follow-up

^a^Two participants did not respond

^b^significant p< .001

## Discussion

Serious digital gaming/gamification was an effective mode of delivery to enhance the understanding and perceptions of ‘players’, who demonstrated a significant improvement of individual attitudes towards dementia. Those playing the game developed a significantly greater optimistic view of the abilities and capabilities of people with dementia. They were also significantly more likely to recognise people with dementia as unique individuals with the same values as any other person. The fact that we see improvements in attitudes is very meaningful, the large effect size shows that there is practical significance of completing the short dementia game. Not only does it attempt to challenge misconceptions about dementia but can then potentially be a tool to start a conversation about dementia.

In this study whether or not the respondent either knew or worked with someone with dementia or received formal training about dementia still were shown to have a significant improvement in their attitude towards dementia. Indeed many young adults may know a person living with dementia but this does not directly mean that they fully understand the condition and how to engage [6]. Similarly, De Vries et al [19] found that specialist dementia training in health care workers in New Zealand made no difference to respondent ADQ scores. A cross-sectional survey conducted by Rosato et al [20] examined factors linked with public knowledge of and attitudes towards dementia. Analysis of 1211 responses demonstrated that despite public campaigns increasing awareness, they reported a high prevalence of personal aversion to dementia which was inversely linked with knowledge and contact. Additionally, significant control over individuals diagnosed with dementia was perceived as necessary even with early stage dementia. They highlighted that knowledge and personal contact do not necessarily translate to specifically supportive responses. As such these attitudes have the potential to escalate to stigma and misconceptions about dementia. In a recent systematic review of the literature on stigma and dementia, Nguyen and Li, 2020 found that participants across studies demonstrated limited knowledge, negative beliefs and emotions such as fear associated with dementia. We know that the promotion of behaviour change and attitudes towards dementia is challenging [21], in particular when the general public only have a moderate understanding of dementia [22], hence effective strategies to facilitate greater social inclusion for people with dementia is a recognised priority [20].

In this study the content of the dementia game was determined by people living with dementia through the sharing of their personal experiences [23]. Co-design allowed *their* priorities of misconceptions to be directly addressed and to be disseminated through a short digital game. If we consider gamification in a pedagogical context, it provides a credible alternative to traditional teaching methods [24]. Serious digital gaming such as www.dementiagame.com provides a fresh approach to learning and enhancing understanding, one which encourages engagement, motivation, and the promotion of learning and problem solving skills [25]. Advocates of digital games value their accessibility and convenience [26]. If the game takes a relatively short time to complete, it can encourage multiple plays and ultimately could reach a wide population [27, 28]. As demonstrated in this study a large number of participants were recruited in a short time period through social media, and due to the recruitment strategy it is highly likely that they were either undergraduate or postgraduate students, the majority being aged 18–34 as would be expected for this population [29]. Hence it could be argued then whether the digital game would be appropriate for, and also reach an older demographic not within academia. Nonetheless, a significant number of older adults now embrace smart and social technology to socialise, to connect and to educate [30], and following the impact of COVID-19, the information technology skills have advanced for many, with online mediums being accessed and used by a record number of people [31].

This study has shown the impact of the prototype of a short dementia game on the public. Nonetheless, future research will be needed to test the effectiveness of the Dementia Game in a full Randomised Control Trial (RCT). In order to reach a more diverse population the provision of different ‘gaming levels’ to address perceptions of the general public, family carers, and healthcare professionals towards dementia is recommended. Although the game has been demonstrated for adults, there is potential to amend the format to educate the younger population using such a novel approach. Due to the online medium of the game the format could also be replicated in other countries including low and middle income counties to help to address public attitudes to dementia [22].

## Strengths and limitations

Recruitment to the study using social media was particularly successful. Despite the large number of respondents who did not complete the follow-up ADQ, the study sample size was still significantly greater than that determined by the power calculation of 44 matched pairs, instead we achieved 457 matched pairs. Therefore, we have very strong evidence to indicate any difference in scores was not by chance. Nonetheless, due to the recruitment strategy it is highly likely that the sample reached was largely homogeneous with undergraduate or postgraduate students from one UK university, and only those who were actively following social media, thus limiting generalisability to the larger population. Following completion of data collection, the dementia game is still accessible, and up to August 2021 has been played over 4000 times.

The pretest-postest design does not have a control or comparison group, hence the internal validity is subject to threats, but it was a pragmatic and cost-effective way to examine the study outcomes, and determine an extension of the research to a RCT. As discussed earlier due to the content being co-designed by people living with dementia this strengthens the content validity of the game.

## Conclusions

The use of a serious digital game has demonstrated a significant positive effect on the respondents’ perceptions of dementia. Overall, there was a greater optimistic view of the abilities of people with dementia and what might be achieved by them, and they were more likely to be recognised as unique individuals with the same values as any other person. Digital gaming has the potential to reach a wide audience and is suitable as a tool to enhance understanding of dementia and improve perception, but further research is needed to reach a more diverse population and test as a RCT to provide definitive evidence for use in policy and practice.

## Supporting information

S1 File (SAV)
